# Patients' Perspectives on Antidepressant Discontinuation and the Pharmacists' Role

**DOI:** 10.1111/bcpt.70207

**Published:** 2026-03-01

**Authors:** Samah Bouarfa, Wilma Göttgens, Suzanne A. Ligthart, Otto R. Maarsingh, Marcel J. Kooij, Pierre M. Bet, Jacqueline G. Hugtenburg

**Affiliations:** ^1^ Department of Clinical Pharmacology and Pharmacy Amsterdam University Medical Centre Amsterdam the Netherlands; ^2^ Amsterdam Public Health Research Institute Amsterdam the Netherlands; ^3^ Department of Primary and Community Care Radboud Institute of Health Sciences, Radboud University Medical Centre Nijmegen the Netherlands; ^4^ Department of General Practice Amsterdam UMC Amsterdam the Netherlands; ^5^ Service Pharmacy Koning Amsterdam the Netherlands; ^6^ Foundation Saphia Amsterdam the Netherlands

## Abstract

Antidepressant (AD) discontinuation care asks for tailored support and alignment with patients' expectations, needs and wishes. However, studies on patients' experiences and perspectives regarding the contribution of the pharmacist to AD discontinuation care are limited. The aim was to gain a deeper understanding of patients' perspectives regarding guidance during AD discontinuation and patients' views on the contribution of the pharmacist. A qualitative, explorative study was conducted with 15 semistructured face‐to‐face and video call interviews. Inclusion criteria were age ≥ 18 years and current or past use of ADs. The audiotapes were transcribed verbatim, coded and analysed by two researchers. The following four themes were identified: (1) experiences with ad use and discontinuation, (2) attitudes and behaviour towards AD discontinuation, (3) expectations and perceptions towards AD discontinuation and (4) needs regarding AD discontinuation. False beliefs about ADs and past negative experiences with AD discontinuation shape beliefs of patients that amount to a reluctance to discontinue ADs. The most urgent needs of patients were timely receiving guidance, including clear and relevant information from accessible, knowledgeable and trustable healthcare providers (HCPs). Pharmacists' easy accessibility, pharmacotherapeutic knowledge, expertise and capabilities should be put to use, as they may help meeting patients' needs on AD discontinuation.

## Plain Language Summary

The aim was to gain a deeper understanding of patients‘ perspectives regarding guidance during antidepressant discontinuation and the contribution of pharmacists. A qualitative study with 15 interviews with current or past antidepressant users revealed four themes related to antidepressant discontinuation: experiences with antidepressant use, attitudes and behaviour, expectations and perceptions, as well as needs. False beliefs about antidepressants and negative experiences with discontinuation shape patients‘ beliefs, amounting to reluctance towards antidepressant discontinuation. The most urgent needs of patients were timely receiving guidance, including clear and relevant information. Pharmacists, as easily accessible and knowledgeable healthcare providers, may help meeting patients‘ needs on antidepressant discontinuation.

## 1 Introduction

Long‐term use of antidepressants (ADs) has been steadily increasing globally [1, 2, 3, 4, 5, 6]. Use of ADs can cause side effects like emotional numbness, weight gain, sexual problems and feelings of dependency [7]. While continuation is advised and indicated in a subgroup of these patients, other patients achieved stable remission and have the indication to discontinue [8]. Patients who are eligible and willing to discontinue ADs are advised to gradually reduce rather than abruptly stop AD use [9]. Whereas discontinuation symptoms may arise in both cases, patients who stop abruptly seem to be more likely to experience discontinuation symptoms [10] or a relapse [8]. In several countries, clinical guidelines recommend to discontinue ADs for 4 or 6–12 months after a single episode of major depression [11, 12, 13]. However, most of these do not specify how to do this. Healthcare providers (HCPs) point out that providing guidance during AD discontinuation is a complex and time‐consuming task [14, 15].

Factors influencing the provision of care for patients discontinuing AD have previously been investigated [16, 17]. Healthcare providers (HCPs) were motivated to support long‐term users in their attempts to discontinue medication in case of possible overtreatment and were aware that AD discontinuation is associated with a certain risk of recurrence [16, 17]. However, the lack of practical support and tools as well as poor professional collaboration appeared clear barriers for adequate AD discontinuation care [17]. In addition, HCPs are not confident about how to deal with patients' fear of discontinuation symptoms and recurrence of symptoms [16, 17, 18]. Protocols, tools and training on communication should be developed to overcome these barriers and to increase the implementation of discontinuation AD care [17]. In this respect, promising effects of the involvement of pharmacists and their role in pharmacotherapeutic treatment of depression have been described [19].

Pharmacists are a common point of contact for many patients and perceived as easily accessible HCPs [20]. The regular contact moments between pharmacists and the patients create opportunities to identify patients eligible for discontinuation and support patients by educating them about discontinuation symptoms and their management. In addition, pharmacists could explain tapering plans and how to adjust them in the case of the occurrence of discontinuation symptoms [21]. This support should align with the expectations, needs and wishes of patients. MeiSSner and colleagues recommended discontinuing within a structured framework providing information and support, which should be individualized and tailored to experiences such as previous discontinuation attempts [22]. Barriers such as feeling dependent on ad, failed discontinuation, lack of side effects, fear of recurrence of symptoms or discontinuation symptoms and social pressure to continue should be taken into account [18, 22, 23, 24]. However, studies on how patients perceive the role of the pharmacist and how these perspectives are informed by the knowledge and expertise of the pharmacist are limited. Guillaumie and coworkers investigated the role of the community pharmacist from patients' perspectives and recommended that pharmacists should provide medication information and support during the whole patient journey from initiating to discontinuing AD in a proactive manner. However, this study did not include patients who were guided by a pharmacist during AD discontinuation [21, 25].

This study is part of the larger Dutch Pharm Guide AD study and aimed to gain a better understanding of patients' perspectives regarding support during AD discontinuation and their views on the contribution of the knowledge and expertise of the pharmacist. Within the Pharm Guide AD study, guidance for support of discontinuation of AD by pharmacists and a protocol for collaboration and agreements between HCPs are developed and evaluated.

## 2 Methods

### 2.1 Study Design

The study was conducted in accordance with the Basic & Clinical Pharmacology & Toxicology policy for experimental and clinical studies [26]. This study was a qualitative, explorative study, in which semistructured face‐to‐face and online interviews were conducted with participants who use or used ADs. These interviews were conducted from December 2022 to January 2023. During these interviews, patients' experiences, expectations and needs regarding AD discontinuations were discussed.

### 2.2 Study Setting

In the Netherlands, pharmacists provide pharmacotherapeutic care according to the guidelines of the Dutch Royal Association of Pharmacists. This includes, among others, the provision of medication information and the performance of clinical medication reviews and consultations. More than 90% of Dutch CPs and GPs attend pharmacotherapeutic audit meetings, which have evolved into an interprofessional education and collaboration activity to improve pharmacotherapeutic policy by making agreements about the prescribing of medication at the population level.

Patients were recruited in the period from November 2022 to January 2023 from pharmacies in the Dutch regions Amstelland and Nijmegen by researchers J.H. and W.G. Patients who visited the pharmacy for repeat prescriptions and used ADs at this time and those who were selected based on previous use according to the pharmacy's medication file were invited by telephone to participate in this study face‐to‐face in the pharmacy or by a video call. A purposive sample of participants was obtained based on their age, gender, duration of treatment and present or past use of ad.

### 2.3 Recruitment

Patients older than 18 years with current or past AD use were called by the pharmacists of their local pharmacy to gauge their interest in participation in this study. After verbal consent, their phone numbers and e‐mail addresses were shared with the researchers. The researchers called the patients to invite them for an interview in the pharmacy or by a video call and sent them information about the research over e‐mail, including a consent statement. The interview was planned at least 1 week after the initial call, and informed consent was signed by the patient and researcher prior to the interview.

### 2.4 Interview Guide

An interview guide to conduct the interviews was developed using Qualitative Methods for Health Research by Green and Thorogood (2018) [27] (Appendix A). Questions were formulated by the research team, after which additional questions were added based on the knowledge and experience of expert pharmacists. The resulting topic list was reviewed by a former AD user to ensure appropriate formulation of sentences and comprehensiveness. In total, four topics were discussed, that is, (I) SSRI pharmaceutical history and treatment was addressed to understand the patient as expert‐by‐experience; (II) AD treatment gave insight into how the patient shapes his/her belief about ADs and all of its aspects; (III) previous discontinuation processes were discussed to assess the experiences of patients; and (IV) guidance and support during AD discontinuation to investigate the experiences of the status quo.

### 2.5 Data Collection

The interviewers were trained in the background of the study and the performance of interviews. The interviewer explained the purpose, duration and confidentiality of the interview, emphasizing that there are no right or wrong answers and that the purpose was to understand their experiences and perspectives. Explicitly, it was asked whether the patient agreed to also touch on sensitive topics (such as side effects, quitting, pressure from environment). The interview started with very open‐ended questions, followed by probing through summarizing and rephrasing and open‐ended follow‐up questions making vague terms concrete. Closed or leading questions were avoided or only used late to clarify. During the conversation, the interviewer aimed to explore experiences in the timeline of medication use with special emphasis on the experiences surrounding discontinuing medication and the interactions with HCP’.

In total, 15 interviews were conducted of which *n* = 14 were face‐to‐face in private rooms of the pharmacies and *n* = 1 was online. No one else was present during the interviews besides the interviewer and the participant. The average duration of the interviews was 56 min (31–80 min). All participants spoke Dutch. After receiving a signed informed consent, the interviews were audiotaped. The research team is presented in Appendix B.

### 2.6 Data Analysis

Data analysis was conducted following the guidelines developed by Braun and Clarke. Braun, V., & Clarke, V. (2006) [28]. First, the audiotapes were transcribed verbatim by two research students and SB. All transcripts were coded inductively and analysed by one research student and SB using MAXQDA.

Transcripts were read closely, and the first five interviews were independently open coded by two researchers (1 research student and SB). The codes were then discussed with the research team (W.G., J.H. and S.B.) until consensus on the codes was reached. All codes were subsequently clustered into an initial coding tree. Remaining data were then open coded to find new codes. These codes were then clustered into themes and subthemes. All data were re‐read, and three discussions between researchers on codes, themes and subthemes using MS Teams took place.

### 2.7 Ethical Approval and Check of Reporting

This study was approved by the Medical Ethics Review Committee of the Amsterdam UMC, location VUmc. The ‘Consolidated Criteria for Reporting Qualitative Research’ (COREQ) checklist was used to ensure qualified reporting (Appendix C) [29].

## 3 Results

### 3.1 Patient Characteristics

In total, 15 participants were interviewed. Their characteristics are presented in Table 1.

**TABLE 1:** Patient characteristics.

Participant number	Gender	Age (years)	Used AD	Duration of AD usage	Therapy status	Time since discontinuation	HCP providing guidance during discontinuation				
							GP	Pharmacist	Psychiatrist	None	Unknown
R1	Male	73	Clomipramine	25 years	Past user	2 years		●		●●	
R2	Female	56	Trazodone and bupropion	13 years	Past user	6 weeks		●			
R3	Female	74	Paroxetine	10 years	Current user	N/A				●●	
R4	Female	24	Citalopram	1.5 years	Past user	8 months				●	●●●
R5	Female	64	Sertraline	4 months	Past user	6 months		●			
R6	Female	68	Paroxetine	30–35 years	Current user	N/A	●				
R7	Female	47	Paroxetine	1.5 years	Current user^a^	2 months		●		●	
R8	Female	52	Venlafaxine	18 years	Past user	Unknown	●●	●^b^			●●^c^
R9	Male	44	Venlafaxine	8–9 years	Current user	N/A	●				
R10	Male	30	Sertraline	8 years	Current user^a^	Unknown	●●		●		
R11	Male	47	Sertraline	4 years	Past user	2 weeks				●	
R12	Male	76	Citalopram	3–3.5 years	Current user	N/A					
R13	Male	46	Escitalopram	4–5 years	Current user^a^	1.5 months				●●	
R14	Male	37	Citalopram	15 years	Current user	N/A					
R15	Female	64	Sertraline	8 years	Current user	N/A					

*Note:* Every ● stands for one AD discontinuation process.

Abbreviations: AD, antidepressant; GP, general practitioner.

^a^ Participants were still discontinuing at time of the interview.

^b^ In collaboration with the GP.

^c^ Discontinuation due to pregnancy.

### 3.2 Themes

The last four interviews did not show any new topics and therefore data saturation was observed. The following four overarching themes were identified from the viewpoint of the participants: (1) experiences with AD use and discontinuation, (2) attitudes and behaviour towards AD discontinuation, (3) expectations and perceptions towards AD discontinuation and (4) needs regarding AD discontinuation care. All themes are recurrent before, during and after discontinuation.

#### 3.2.1 Experiences With AD Use and Discontinuation

##### 3.2.1.1 After Start

Most participants were not informed about discontinuation and wished to have received this information from the start.

*‘Again, you get that stuff, and if you don't say anything you won't get rid of it for the rest of your life. There is not a person on earth who will say to you, eh, they won't even stop. At least they've never said that to me. Not a human…’* (Respondent 1 [R1]).

Participants who recently started ADs seem to be more informed about discontinuation at start compared to participants who started ADs a longer time ago. The latter group was more convinced of lifelong AD use.

During continued AD use, the medication's effectiveness was seldom evaluated. Experiences with side effects motivated patients to consider stopping the medication.

Motives to discontinue were various: to think more clearly, not be able to drive, ineffectiveness of the ad, flattened emotions, nudged by family, questioning the purpose, no motivation for adherence, fear for side effects One participant explained that using ADs helped to avoid dips, but also tempered the highlights of life.

*‘Well, I've always been someone who experiences very high highs and very low lows […] I no longer had those very high highs, but also not those very low lows’* (R8).

Like her, some participants did not anticipate to experience numbness.

*‘You have more feelings, when I look back now at how I am now and how I was, I think you are, if you swallow that, a very dull person, in terms of emotions’* (R1).

##### 3.2.1.2 During Discontinuation

While some participants preferred guidance by GPs because of acquaintance, others preferred guidance by a pharmacist. From their point of view, the GP seemed to have too little time.

*‘I also had a lot of confidence in it because she [pharmacist] really looks at what it does. If you are at the doctor's office, 10 minutes, sure, fine, but he sees you, he will ask how you are doing, and before you know the 10 minutes are over. Yes, but it is not the information you get from a pharmacist or someone who knows about it. I really enjoyed it’* (R5).

Accessibility also seemed to be an important factor.

*‘And I think that if it didn't work for the GPs, I would contact [pharmacist], because he is easily accessible, even better to reach than my own GP’* (R7).

Others felt like that the GP knew them better. Some participants did not care who provided AD discontinuation care as long as the HCP was knowledgeable, experienced, reflective and emotionally balanced in psycho‐pharmacotherapeutic practice.

‘*Well, actually, some insight into the process, a sense of the process. I also think a certain level of firmness is essential, a background in psychology so you have a sense of what you're dealing with’* (R2).

Either way, several factors seemed to increase participants' confidence to discontinue. Examples are a tailored program, contact moments every 1–4 weeks, living calm periods and easy accessibility and dose increase if necessary. Some participants thought that guidance is necessary and the majority of participants thought that it was an important precondition to discontinue safely. Participants who were guided by the pharmacist thought the pharmacist provided them with the best information possible.

‘*No, I can recommend the tapering process I went through with [pharmacist] to everyone, once again it was truly fantastic’* (R1).

For some participants, slow discontinuation over a longer period of time worked better.

##### 3.2.1.3 After Discontinuation

After discontinuation, some participants felt like their ‘old’ self again, but at the same time they were on guard for recurrences of anxiety or depression. While some participants had no symptoms after stopping, others felt like that they became emotionally less stable or could not sleep well anymore. Because of the patient's increased emotional sensitivity, in some cases, the family of a participant urged to restart the AD again, while the participant thought that it was not appropriate.

#### 3.2.2 Attitudes and Behaviour Towards AD Discontinuation

For some people, AD use had become a habit, and discontinuation was not considered unless someone asked about it. When it came to decision‐making, family and friends could have significant influence, causing acceptance or aversion to AD discontinuation. Common reasons to start AD discontinuation from the participants' perspective included medication aversion, numbness and acquaintance with temporary use. A major reason to delay AD discontinuation was patients getting confused and hesitating to choose, torn between fears for withdrawal and relapse and fears for dependence from prolonged AD use.

*‘Yes, and I wasn't allowed to just stop, and I didn't think that was the solution either, because that [idea of stopping] also scared me again’* (R4).

In addition, some patients were convinced of the need for lifelong AD use. This was mainly based on the perception that serotonin was lacking in the brain and needed to be enhanced by ADs.

Some participants appreciated people talking about AD discontinuation. Whereas some participants chose to inform family or relatives about their medication use, other participants chose not to because they did not feel understood or they were afraid that people would judge them. One participant did not inform her relatives about her AD use until a certain moment.

*‘Yes, in the media, such as television talk shows you hear: yes, I also had panic attacks. And then I think oh okay I wouldn't have dared to tell that before and now it's all open’* (R6).

Discussing AD use with HCPs and feeling misunderstood could cause worsening of symptoms from the viewpoint of the participant.

*‘Yes, then you go even deeper, because you're not going to talk about it then’* (R6).

One patient even explained that he would not start AD discontinuation if he was not taken seriously by his HCP.

*‘What I'm saying is, I got the feeling he really took it seriously and didn't downgrade* *(my feelings)* *like, “Yeah, it's no big deal”’* (R10).

The willingness to discontinue largely varied between participants. Whereas one participant believes navigating life without medication is possible, another participant avoids every possibility to experience a negative feeling that resembles the symptoms of anxiety or depression.

#### 3.2.3 Expectations and Perceptions Towards AD Discontinuation

##### 3.2.3.1 Expectations and Underlying Beliefs

Participants believed that there were a number of preconditions to discontinue ADs successfully: using small steps, discontinuation schedules, having a trusted person in the social context and guidance. However, most participants believed that not all HCPs were able to provide guidance. The shortage of time, perceived lack of specific knowledge, transparent communication, practical support and interdisciplinary collaboration were key factors undermining patient confidence in healthcare professionals for AD discontinuation guidance.

After stopping ADs, two regrets were common among most participants. They regretted reading information on social platforms on the internet and consulting other participants' experiences because this increased fear and anxiety. Online discussions and patients' dominant discontinuation narratives emphasized severe withdrawal symptoms and negative experiences, which created fear or anticipation of suffering. This ‘nocebo effect’ (expecting harm and thus experiencing it more intensely) made discontinuation feel more daunting than it might have been otherwise.

Second, some participants regretted not starting earlier with AD discontinuation. Participants expected GPs to address AD discontinuation at an earlier stage. Looking back, some participants also did not anticipate flattening of emotions to occur after long‐term AD use. Some participants believed ADs were addictive and were prescribed or restarted too easily and even said that they did not agree to do so. Some even said that the ADs were imposed upon them. On the other hand, most participants understood that it was necessary at the time.

##### 3.2.3.2 Perspectives

Some participants questioned long‐term AD use, while others thought that periods of mental stability required continuation of AD use. In this line of reasoning, one idea seemed to be common.

*‘After all, I am the boss of my own body, if I say it is okay, I want to get rid of it, then of course I can get rid of it’* (R3).

In order to start AD discontinuation, participants thought there should be a trusting relationship between the participant and HCP. A referral from the GP to a counsellor also felt trustworthy.

*‘Just like what my GP did, she referred me to [pharmacist]’* (R8).

Some participants thought that the GP is closer to the patient than the pharmacist. Some participants were unfamiliar with the pharmacists' capabilities to provide AD discontinuation care.

*‘Well, if I had another GP who hadn't had [pharmacist], then I don't know how it would have gone.’* (R2).

Some people chose to discontinue autonomously. However, reflecting on the discontinuation process, most participants think that this could have gone wrong. Some participants think that their motivation and commitment was key for successful discontinuation. Most participants were content that AD use has stopped and thought it was nice to feel independent from ADs . The minority of participants were generally negative about ADs and described them as ‘trash.’

#### 3.2.4 Needs Regarding AD Discontinuation Care

##### 3.2.4.1 A Specialized and Easy Accessible Person as Counsellor and Therapeutic Relationship

There was a clear need for a specialized person as counsellor with certain competences: personal contacts, good accessibility, firmness and maturity, background in psychiatry/psychology, pharmacology and pharmacotherapeutic knowledge and expertise.

*‘The GP is fine, but it is general. I think that with this type of medication, a little more knowledge and ability [is needed], also because not everybody reacts the same [on the drug]*’(R5).

If the pharmacist provided the AD discontinuation care, patients thought that the GP needed to be involved in case of emergency.

In addition, participants considered a therapeutic relationship very important and addressed the continuity of care, as well as regular contact moments. One participant expressed missing support from the HCP at crucial moments.

*‘She was not there again, or she was only there for a day, and then she could not call. Then I was in really deep shit. I then spent 2 weeks at home and completely lost it, and I was actually just a danger to myself’* (R4).

Easy accessibility from the HCPs was a recurrent theme and many participants opted for regular contact moments, not only during AD discontinuation, but also before.

*‘Yes, and that I don't have to wait for years or months before someone contacts me’* (R4).

Most participants would appreciate receiving guidance from time to time, even before AD discontinuation.

##### 3.2.4.2 Information About Medication and Review of Medication Use

There was also a clear need for information about side effects, duration of use and what to expect during therapy and discontinuation. One participant opted no need for extensive information as long as the medication was effective and described himself to be ‘lazy.’

Most participants needed the HCPs to review their medication use and to raise the question to discontinue. Some were more motivated to do so if there was an option to discontinue at a slow pace. Few participants thought the initiative to discontinue lay with the patient (Table 2).

**TABLE 2:** Themes and subthemes.

Themes	Subthemes
Experiences with AD use and discontinuation	
Before discontinuation	Not being informed about discontinuation from the start. Being more convinced of lifelong AD use when starting AD a longer time ago. Medication's effectiveness was seldom evaluated. Experiences with side effects drives considering stopping the medication. Motives to discontinue: to think more clearly, not be able to drive, ineffectiveness of the ad, flattened emotions, nudged by family, questioning the purpose, no motivation for adherence, fear for side effects. Not anticipating to experience numbness.
During discontinuation	Preferred guidance by GPs because of acquaintance, but often lacks time. Preferred guidance by a pharmacist because of specific knowledge and accessibility. No preference for specific HCP as long as HPC was knowledgeable, reflective, emotionally balanced and experienced in psycho‐pharmacotherapeutic practice. Increase confidence to discontinue if access to: a tailored program, regular contact moments, living calm period, flexible dose regime. Guidance is necessary and an important precondition to discontinue safely. Convinced by experience that the pharmacist provides the best information possible.
Attitudes and behaviour towards AD discontinuation	
Before discontinuation	Habituation of AD use and not considering discontinuation unless been asked about it Family and friends influence decision‐making causing acceptance or aversion to AD discontinuation Reasons to start AD discontinuation included medication aversion, numbness, informed about temporary use. Major reason to delay AD discontinuation was getting confused, hesitating to choose, torn between fears for withdrawal and relapse and fears for dependence from prolonged antidepressant use. Being convinced of the need for lifelong AD use based on the perception that serotonin was lacking in the brain and needed to be enhanced by ADs . Appreciation to talking about AD with family or relatives discontinuation varies with the degree in which they feel understood or, on the contrary, feared to be judged. Feeling misunderstood by the HPC could cause worsening of symptoms Willingness to discontinue largely varied from a strong believe that navigating life without. Medication is possible, to avoiding every possibility of experience negative feelings that resembles the symptoms of anxiety or depression.
Expectations and perceptions towards AD discontinuation	
Expectations and underlying beliefs	Preconditions to discontinue ads successfully: using small steps, discontinuation schedules, having a trusted person in the social context and professional guidance.
During discontinuation	Key factors undermining patient confidence in healthcare professionals were: shortage of time, perceived lack of specific knowledge, transparent communication, practical support and interdisciplinary collaboration were.
After discontinuation	Regretting reading information on social platforms on the internet and consulting other participants' experiences because this increased fear and anxiety. This ‘nocebo effect’ made discontinuation feel more daunting than it might have been otherwise. Regretting not starting earlier with AD discontinuation due to not being informed about the possibility of discontinuation in an earlier stage. Not anticipating flattening of emotions to occur after long‐term AD use. Being prescribed ad's or restarting too easily even if they did not agree to do so. Experience of ADs being imposed on one self. Questioning long‐term AD use or, on the contrary, thinking that periods of mental stability requires continuation of AD use. Anticipating autonomy in decision making about discontinuation. A trusting relationship between the participant and HCP In order to start AD discontinuation. A referral from the GP to a counsellor also felt trustworthy—thinking that the GP is closer to the patient than the pharmacist. Being unfamiliar with the pharmacists' capabilities to provide AD discontinuation care—choosing to discontinue autonomously but when reflecting on the discontinuation process, thinking this could have gone wrong. Motivation and commitment was key for successful discontinuation. Being content that AD use has stopped and enjoying to feel independent from ADs .
Needs regarding AD discontinuation care	
General	A decisive need for a specialized person as counsellor with certain competences: personal contacts, accessible, firm and mature expertise, background in psychiatry or psychology, pharmacology and pharmacotherapeutic knowledge and expertise.
Before discontinuation	Easy accessibility and regular contact moments, before and during AD discontinuation. Appreciate receiving guidance from time to time, even before AD discontinuation. Receiving review of medication use and sound and timely information about: side effects, duration of use and what to expect during therapy and discontinuation. Needing the HCPs to review their medication use and raise the question to discontinue. Being motivated to discontinue when having an option to discontinue in a slow pace.
During discontinuation	Needing the GP to be involved in case of emergency if the pharmacist guides AD discontinuation. Considering a therapeutic relationship and continuity of care very important. Preference for face‐to‐face contact or telephone calls or online communication varies ‐valuing a discontinuation schedule. Appreciating flexibility in the process to speed up or slow down the pace of lowering the dose.

##### 3.2.4.3 Guidance of AD Discontinuation

Most participants needed guidance to discontinue.

*‘You really need someone to stop that AD’* (R1).

Whereas some patients prefer face‐to‐face contact moments, others explicitly addressed the need for telephone calls or online communication through chats. Either way, there was a need to share experiences and expectations with HCPs. Also, most participants valued a discontinuation schedule. One patient commented.

*‘I think it is important that you have a schedule for tapering off, that it explains that you can take 2 pills today, and 2 more tomorrow, but the next day you will take 1 3/4 because not all the medicines can be tapered off using droplets’* (R5).

Patients appreciate flexibility in this process, for instance if HCPs would propose:

*‘We can also speed things up if you think you can actually take steps more quickly. We can also slow things down a bit, so we stay at a certain dose longer. So there's a kind of, well, a plan for what to expect, and we can adjust it along the way if necessary. Yes, I'd like that’* (R9).

## 4 Discussion

This study is part of the Pharm Guide AD study and aimed to gain a better understanding of patients' perspectives regarding guidance during AD discontinuation and their views on the contribution of the knowledge and expertise of the pharmacist. During this qualitative, explorative study among 15 patients, we identified the following four themes: (1) experiences with AD use and discontinuation, (2) attitudes and behaviour towards AD discontinuation, (3) expectations and perceptions towards AD discontinuation and (4) needs regarding AD discontinuation. Our study found that the majority of participants require guidance during AD discontinuation. Most of them expect HCPs to take the initiative to start this process. This finding is in line with other studies [23]. One of the most recurrent themes after the start of the AD discontinuation process is the role of the HCPs in the AD discontinuation process. The two most recurrent subthemes were availability and knowledge/expertise.

In terms of availability, participants valued a HCP who (a) has enough time to provide AD discontinuation care and (b) is easily accessible. In other studies, participants have also described their ideal counsellor as easily accessible [30]. Some participants thought GPs are not as easily accessible as pharmacists. In addition, some participants felt like that GPs are limited in their time spent per consultation. AD discontinuation care could be facilitated more to the need of these patients by a more efficient division of responsibilities between pharmacists and GPs or psychiatrists.

In addition, this could also help initiate a structural pharmacy‐based review of long‐term AD use, as most patients expect HCPs to start the conversation about AD discontinuation. Over the last few years, the work pressure for GPs has risen, and GPs seem to be reluctant to actively start an extensive task like AD discontinuation. Therefore, an integrated form of collaboration, in which GPs refer patients to a community pharmacist, might be an efficient solution [17]. In our study, we found that patients who do not have any experience with pharmacists as counsellor are hesitant towards the idea of a pharmacist as counsellor. However, the experiences of patients who were guided by the pharmacist expressed positive feedback and gratitude. Visibility of the pharmacist as counsellor in this context can be improved, for instance, by referrals from GPs to pharmacists.

These referrals seem to be key to initiating a trusting relationship between pharmacists and patients. On the other hand, some patients directly contacted the pharmacist about their interest in AD discontinuation and started the discontinuation process after the pharmacist requested the AD discontinuation prescription from the GP. These findings demonstrate that in some settings the role of the pharmacist in AD discontinuation care already exists. However, at the moment this cannot be deemed as usual care. Education may help unexperienced pharmacists to overcome challenges like discussing taboos around ADs and fear of risks associated with AD discontinuation [31]

Trazining in communication techniques is also required, as AD users often hesitate to discuss their medication due to pervasive stigma and fear of judgement. Based on our results, HCPs should avoid misunderstanding the patient to maintain patients' trust in the discontinuation process. Downgrading feelings seems to work counterproductively, whereas listening carefully and attentively enhances the trust in the provider‐patient relationship and facilitates shared‐decision making. These findings are consistent with the research of Morant et al. [32] and of Grim et al. [33].

Other studies confirm that participants attribute competences to their ideal counsellor and expect them to show empathy, to be supportive, professional and most importantly knowledgeable [29].

Therefore, applying communication techniques with the existing pharmacotherapeutic knowledge and expertise could help meet patients' needs and provide them with information that is necessary to discontinue ADs.

More specifically, this may help overcome and manage fear to discontinue and fear of discontinuation symptoms or to rectify false beliefs about AD use, like the addictive nature of ADs or a missing substance in the brain [34]. It is demonstrated that patients' concerns about severe discontinuation symptoms persist as the result of poor information provision on DS, which undermined their actions to discontinue [7]. Discontinuation symptoms can be severe and prolonged and may cause uncertainty and fear of discontinuation, which may fuel the desire of patients to continue treatment. On the other hand, false beliefs, such as assuming that ADs should be used life‐long, also contribute to long‐term AD use as the result of lack of information or false information. HCPs should provide clear and relevant information before and during the AD discontinuation process to resolve potential false beliefs. This requires training of HCPs.

## 5 Strengths and Limitations

The qualitative methods applied yielded information regarding the views and opinions of (former) AD users on the pharmacists' role during AD discontinuation and opportunities to improve AD discontinuation care. Saturation was reached after analysis of 11 interviews. A strength of the study is that we interviewed a random mix of patients from several pharmacies in two different Dutch regions. Participants' characteristics varied in gender (7 male/8 female), age (24–76 years), AD used, duration of AD use (4 months‐35 years) and prior experiences with AD discontinuation. Some participants have never discontinued and others have had multiple prior discontinuation attempts. In addition, the counselling HCPs indicate that the group was diverse, representing varying categories of patients. Some participants started ADs or had prior discontinuation attempts years ago.

Recall bias could have affected the ability of participants to reflect on experiences. Several interviews were performed using a video call. This may have reduced their responsiveness in case other people were in the room. On the other hand, in their own home, a participant may feel more comfortable and experience less distraction by a strange surrounding. In addition, the quotes were translated from Dutch into English. All translations were evaluated by the research team.

## 6 Conclusions

The results of this study shed light on the potential contribution of pharmacists to patient education and support during AD discontinuation. It also provides insight into what patients consider important and what is needed to implement this approach.

Negative experiences with AD discontinuation and false beliefs about ADs seem to shape the beliefs of patients that contribute to a reluctance to discontinue. The most urgent needs of patients were timely reception of clear and relevant information from HCPs to cope with fear and resolve false beliefs. There is a high need for guidance by a trusted HCP during AD discontinuation. Pharmacists' easy accessibility, pharmacotherapeutic knowledge, expertise and capabilities should be put to use, as they may provide the patient with relevant information and help meet patients' needs on AD discontinuation. Training of pharmacists should be considered.

## Funding

This work was supported by Stichting MAG and ZonMw (10100162210006). These sponsors had no role in the study design, data collection, analysis, data interpretation, writing process or any decision to submit the article for publication.

## Conflicts of Interest

The authors declare no conflicts of interest.

## Data Availability

The data that support the findings of this study are available on request from the corresponding author. The data are not publicly available due to privacy or ethical restrictions.

## Appendix A A. Questionnaire

I. Pharmaceutical history SSRI and treatment (to understand the patient as an expert‐by‐experience)

1. Pharmaceutical history SSRI

a. Do you remember the reason why this medication was prescribed?

b. Can you explain how using this drug has worked out?

i. Do you have any idea why the decision was made for you to use this drug?

2. Treatment

a. What agreements were made regarding the use of this drug?

i. Were those agreements sufficient? (ask about experiences, concrete events etc.)

ii. What happened in the pharmacy? And in the GP office?

iii. Who did you speak with?

iv. Was the duration of use clear?

v. What information regarding discontinuation did you receive before starting the treatment?

b. Did you know what you could expect from the treatment?

i. What were your expectations of the treatment?

ii. On a scale of 1–10: how effective was this drug for you?

iii. What effects of the drug did you notice?

c. Did you miss any information that you had to look for?

i. Did you have to consult the internet?

d. Did you have any form of social support?

i. Is there someone who helps you with the medication?

ii. What role does your circle of friends have in the treatment?

iii. What role does your family have in this treatment?

e. Can you tell me about other treatments that you have received?

i. For example CGT?

3. Is there anything you want to share about your experience with this drug or treatment that we didn't already discuss?

II. Prior discontinuation trajectories

1. Are you currently considering to discontinue?

a. What did you want to achieve with discontinuation?

b. How did you expect the discontinuation process to proceed?

2. What are/were your expectations of discontinuation of drug X?

a. In what way did you think this would go (or do you think this will go)?

b. Wat difficulties did you expect (or do you expect)?

c. What did you expect to go well?

3. Have you tried to discontinue drug X before?

a. If yes, how many times?

b. Can you describe the process for me? From the first conversation about discontinuation until the discontinuation result?

i. What were your experiences?

ii. What went well?

iii. What went not so well? What symptoms did you have?

iv. What made you choose to restart drug X?

c. How did you come up with the idea to discontinue?

d. Why do you think discontinuation succeeded (or did not succeed)?

i. If you could alter the approach, what would you change?

e. How do you view the next discontinuation process?

4. Is there anything you want to share about prior discontinuation trajectories that we didn't already discuss?

III. Guidance and support during discontinuation

1. How do you want to be involved in the decision about discontinuation of drug X?

a. In terms of guidance, what is essential for you to follow your treatment?

2. What do you expect from guidance of health care providers?

a. What do you expect from the GP?

b. What do you expect from the pharmacist?

c. What do you expect from the psychiatrist?

3. According to you, what do you need to discontinue?

a. What tools do you need to discontinue successfully (i.e., discontinuation schedule, app)?

b. How can your relatives (family, friends) help you with discontinuation?

4. What information about discontinuation do you think is important to receive?

a. i.e., about recurrence? Discontinuation symptoms?

b. To whom would you address this?

5. Do you think the pharmacy can have a bigger role to provide guidance during discontinuation of drug X? If yes, in what way do you want them to provide care?

6. Is there anything you want to share about guidance and support during discontinuation that we didn't already discuss?

IV. Personal information

a. How does your life look at the moment?

a. Do you work?

b. Do you have kids?

c. What is your civil status?

d. What is your highest education received?

## Appendix B

Roles and backgrounds

Patients were recruited by W.G. (female) and J.H. (female) from multiple pharmacies in the Netherlands. W.G. is a pharmacist (PharmD) and philosopher (MA) with experience and interest in the field of antidepressant discontinuation. J.G. is a professor in Disease Prevention and Health Promotion, University of Curaçao, associate professor in Pharmacoepidemiology and Pharmaceutical Care, AmsterdamUMC, location VUmc and community pharmacist (PharmD).

Interviewers were SB (female), research students FC (female) and MS (female). All interviewers were trained to interview and made notes during the interviews. Researcher S.B. has a PharmD degree and was a PhD candidate at the time of the interviews. F.C. and M.S. had a BSc degree and were PharmD students at the time of the interviews. All three of them also performed the analysis. S.B. wrote the manuscript, in close collaboration with W.G. and J.H.

P.B. (male), O.M. (male), and S.L. (female) provided content feedback on the manuscript. P.B. is a hospital pharmacist (PharmD, PhD) and clinical pharmacologist with a special interest in psychopharmacology. O.M. is a professor, G.P., researcher and epidemiologist at the Department of General Practice, Amsterdam UMC, location VUmc. One of his research focuses is depression. S.L. is a general practitioner (MD, PhD) with a special interest in the research field.

Relationship with participants

There was no relationship between the interviewers and participants prior to study commencement.
